# Critical role for resource constraints in neural models

**DOI:** 10.3389/fnsys.2014.00154

**Published:** 2014-08-22

**Authors:** James A. Roberts, Kartik K. Iyer, Sampsa Vanhatalo, Michael Breakspear

**Affiliations:** ^1^Systems Neuroscience Group, QIMR Berghofer Medical Research InstituteBrisbane, QLD, Australia; ^2^Faculty of Health Sciences, School of Medicine, University of QueenslandBrisbane, QLD, Australia; ^3^Department Clinical Neurophysiology, Children's Hospital, Helsinki University Central Hospital, University of HelsinkiHelsinki, Finland; ^4^Royal Brisbane and Women's HospitalHerston, QLD, Australia

**Keywords:** criticality, mathematical models, metabolic resources, burst suppression, scale-free dynamics

## Abstract

Criticality has emerged as a leading dynamical candidate for healthy and pathological neuronal activity. At the heart of criticality in neural systems is the need for parameters to be tuned to specific values or for the existence of self-organizing mechanisms. Existing models lack precise physiological descriptions for how the brain maintains its tuning near a critical point. In this paper we argue that a key ingredient missing from the field is a formulation of reciprocal coupling between neural activity and metabolic resources. We propose that the constraint of optimizing the balance between energy use and activity plays a major role in tuning brain states to lie near criticality. Important recent findings aligned with our viewpoint have emerged from analyses of disorders that involve severe metabolic disturbances and alter scale-free properties of brain dynamics, including burst suppression. Moreover, we argue that average shapes of neuronal avalanches are a signature of scale-free activity that offers sharper insights into underlying mechanisms than afforded by traditional analyses of avalanche statistics.

## Introduction

A substantial body of evidence now suggests that the brain operates near criticality. That is, analyses of healthy (Meisel et al., 2013) and pathological (Roberts et al., 2014) brain activity yield parameters lying near the cusp between stability and instability. Such a state confers benefits of increased flexibility (Kinouchi and Copelli, 2006; Shew et al., 2009), optimized information transfer (Beggs and Plenz, 2003; Shew et al., 2011), and increased storage capacity (Haldeman and Beggs, 2005; Shew et al., 2011). However, the question of *how* the brain maintains criticality is not clear. Prevailing theories posit various mechanisms but little attention has been paid to unifying these. In this Perspective Article, we argue that since existing mechanisms ultimately rely on various forms of activity-dependent modulation, models that integrate neuronal activity with metabolic resources present an opportunity for unifying existing theories of neuronal criticality. Moreover, we suggest that disambiguation of competing models would benefit from complementing traditional approaches of calculating scaling exponents with analyses of the deeper scaling properties encoded in average event shapes. This has been employed successfully in physics, but has only recently found traction in neuroscience.

## Competing mechanisms in models of critical brain dynamics

Much of the attention on critical brain dynamics has centered on neuronal avalanches in fluctuating local field potentials measured using small grids of electrodes (Beggs and Plenz, 2003; Petermann et al., 2009; Priesemann et al., 2013), though signatures of criticality have been detected in many other large-scale measurements including MEG (Palva et al., 2013; Shriki et al., 2013), EEG (Linkenkaer-Hansen et al., 2001; Palva et al., 2013; Roberts et al., 2014), and fMRI (Haimovici et al., 2013). Modeling efforts have tended to focus on spatial avalanches in networks of spiking neurons, with relatively few analyses of criticality in large-scale models relevant to EEG (Steyn-Ross et al., 1999; Robinson et al., 2010; Aburn et al., 2012). Such models will be crucial for describing the macroscopic scale accessible in human studies.

Models of critical brain dynamics typically fall into two classes: those with a tuning parameter and those that self-organize. Models with a tuning parameter only exhibit critical dynamics when the model parameters are set precisely at the critical state, such as in branching processes (Beggs and Plenz, 2003; Haldeman and Beggs, 2005) and in typical mean-field models (Steyn-Ross et al., 1999). Parameter-setting mechanisms are outside the scope of these models by design—presumably slow parameter modulations exist to set the parameters but these are not explicitly modeled. In self-organizing models, the parameters evolve “naturally” to the critical point, usually involving synaptic plasticity based on either the strength of activity (De Arcangelis et al., 2006; Levina et al., 2007) or spike timing (Meisel and Gross, 2009; Rubinov et al., 2011). Another means of self-organizing to a critical point is to grow a network from scratch, with activity-dependent plasticity governing the growth rules (Tetzlaff et al., 2010). A common feature of self-organization in physics is a separation of time scales between the slow build-up of energy and fast relaxation or dissipation—earthquakes are a classic example (Sethna et al., 2001). While similar time-scale separations exist in many neural models, an explicit link to energy (or at least a proxy for energy) is rarely made. We argue that such links will be important for unifying various tuning mechanisms.

## Average burst shapes are sensitive to underlying mechanisms

The proliferation of models exhibiting criticality has centered on reproducing scale-free distributions of event sizes and durations seen empirically, with varying degrees of biological realism versus abstraction. While criticality likely arises in more than one context in the brain, it is also likely that there is room to unify theories where they describe the same phenomenon. Conversely, it is important to find ways of distinguishing between competing mechanisms that do not necessarily perform equally well in all settings. Disambiguating competing models is likely hampered by the limited set of measures typically used when benchmarking candidate models against empirical data. Power-law exponents are the most widely used means of testing model validity. However, multiple models can exhibit the same exponents while having different mechanisms and avalanche shapes (Sethna et al., 2001). Thus, average shapes are a sharper test of competing theories—this has been particularly successful in studies of ferromagnetism, where existing theories that reproduced correct exponents were shown to not reproduce the correct shapes (Mehta et al., 2002). By moving beyond traditional analyses, average shapes reveal deeper mechanistic insights (Zapperi et al., 2005; Papanikolaou et al., 2011). This approach has recently been applied in neuroscience revealing a variety of shapes in both data and models (Friedman et al., 2012; Priesemann et al., 2013; Roberts et al., 2014). In particular, invariance of average shapes across time scales is a strong indicator of scale-free dynamics, while a scale-dependent change in shape (such as asymmetry at long time scales, quantified with skewness), hints at deviations from perfect scale-free behavior that may not be visible in typical event statistics such as size distributions.

**Figure 1:**
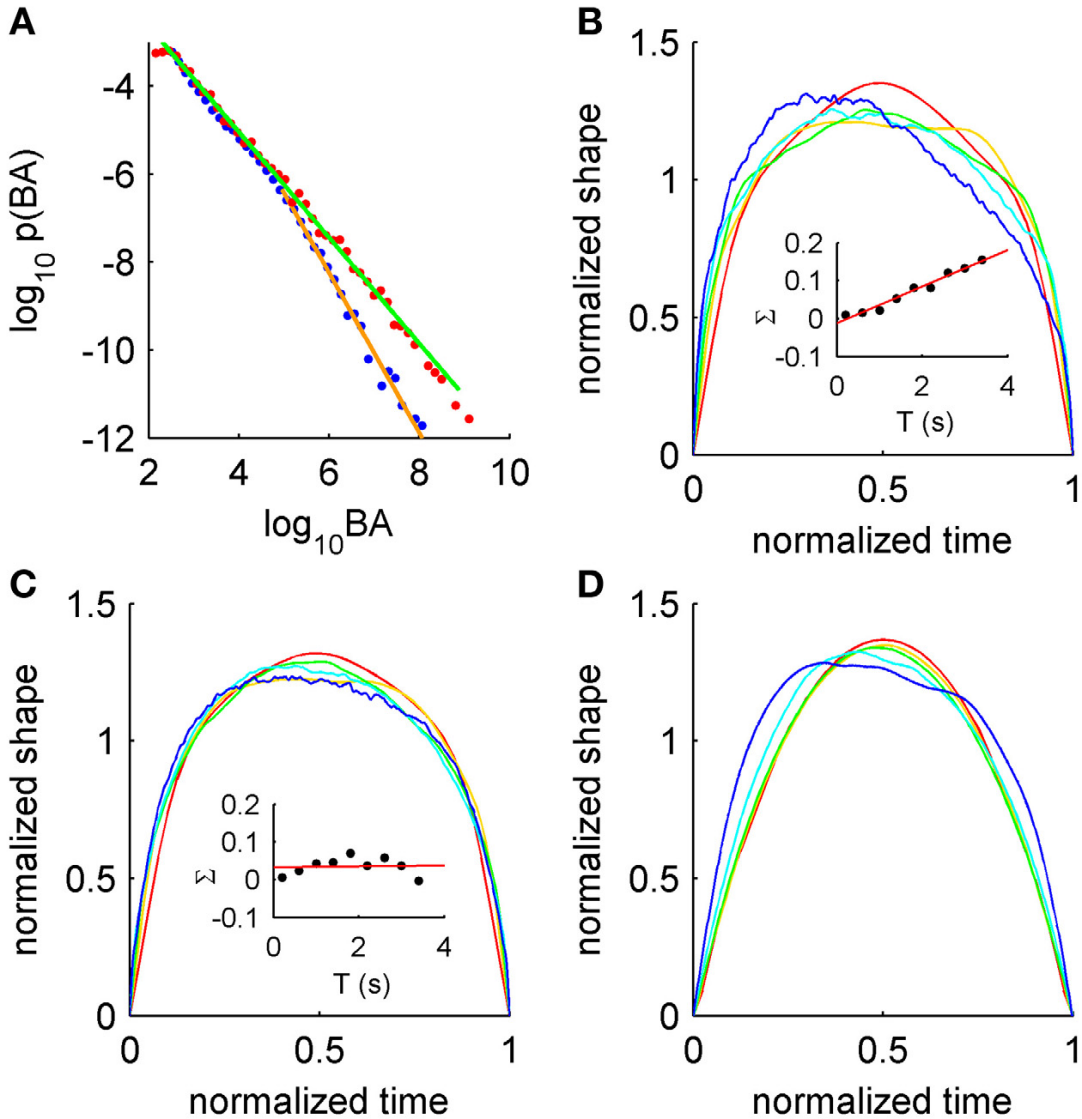
**Signatures of criticality in burst suppression EEG. (A)** Distributions of burst area (BA) for burst suppression (red) and later in recovery (blue), with corresponding power-law fits (green and orange, respectively). **(B)** Asymmetric average burst shapes for burst suppression EEG over a range of durations (red to blue, shortest to longest). Inset: burst skewness (Σ) as a function of duration *T* for burst suppression with linear fit (red). **(C)** Symmetric average burst shapes from EEG recorded later during recovery. Inset: burst skewness later in recovery. **(D)** Asymmetric average burst shapes from the simple model showing resource depletion. For more details see Roberts et al. (2014).

Recently it was shown that burst suppression following hypoxia exhibits a striking example of scale-free dynamics (Roberts et al., 2014). Burst suppression occurs in various abnormal brain states such as recovery from hypoxia and during anesthesia, and is characterized by near-quiescent “suppressed” periods punctuated erratically by large-amplitude “bursts” of electrical activity. In post-hypoxic burst suppression, scale-free properties vary across the recovery period, with scale-free burst distributions prominent during the burst-suppression phase, exhibiting stronger truncation upon the resumption of healthy activity (Figure 1A). These statistical features appear to relate closely to the pathophysiology, as they co-vary significantly with later clinical outcome (Iyer et al., 2014). Since criticality is usually associated with healthy states, existence in neonatal burst suppression thus broadens criticality's applicability to at least one pathology, and suggests that the developing brain may provide a new window into critical brain states.

Power-law scaling is also seen in duration distributions and in the scaling relationship between sizes and durations, with the exponents related in line with theories of crackling noise (Roberts et al., 2014). But scaling exponents do not tell the whole story. Scale-invariance of burst shapes is disrupted in the burst-suppression phase, showing increasing leftward asymmetry at long time scales (Figure 1B). Again, this feature of metabolically-compromised cortex diminishes upon resumption of healthy activity (Figure 1C, and cf. insets of panels **B** and **C**). This scale-free signature is thus acutely sensitive to the pathophysiology. In light of their success in explaining Barkhausen noise in ferromagnetism (Sethna et al., 2001; Mehta et al., 2002; Zapperi et al., 2005), where analysis of average shapes led to the development of new models, we argue that average shapes are under-utilized as a signature of scale-free dynamics in neural systems. We hope that rigorous testing of models against data will enable similar progress to that seen in the study of ferromagnetism. Moreover, analysis of events themselves, rather than coarse summary statistics, is underused in clinical settings.

## Unifying mechanisms of self-organization via biophysical modeling of resource constraints

Asymmetry of the average shape arises from history-dependent effects (Zapperi et al., 2005; Roberts et al., 2014). A well-established example in physics is the response of a ferromagnet in a slowly changing external field (a classic, controllable example of criticality). There, the external field aligns microscopic domains in the magnet, but instead of gradually aligning in unison the individual domains flip suddenly and erratically, triggering similar flips in their neighbors. This yields a bursty signal termed Barkhausen noise, a striking example of crackling noise (Sethna et al., 2001) with characteristic asymmetric burst shapes that lean to the left. These shapes were explained using a model with history dependence derived from the dynamics of domain wall pinning (Zapperi et al., 2005; Papanikolaou et al., 2011). For burst suppression in post-hypoxic neonates, it was found that left-leaning bursts (Figure 1D) arise from a simple model with activity-dependent damping:

(1) $$\dot{x}\;=-\lambda x+\xi \left(t\right),$$

(2) $$\lambda \;=\alpha_{2}\int_{-\infty }^{t}e^{-\alpha_{1}\left(t-\tau \right)}x{\left(\tau \right)}^{2}d\tau .$$

Here, *x* represents neuronal activity, ξ is a Gaussian white noise drive, λ is a damping constant, and *α*_1_ and *α*_2_ are constants. This form was motivated by the fast-out slow-return nature of the leftward asymmetry: damping is low at the beginning of bursts but increases with the increasing activity in its recent history. This is consistent with the post-hypoxic brain being acutely sensitive to its constrained metabolic resources. Although this is a simple phenomenological model, the central idea of activity-dependent modulations is widely applicable. For example, metabolic constraints have recently been incorporated into a cellular model to explain a different (non-scale-free) type of burst suppression induced in adult EEG during propofol anesthesia (Ching et al., 2012). Moreover, advancing technologies for measuring metabolic variables will yield rich data sets prompting model development. Oxygen availability has recently been shown to be tightly coupled to levels of excitability in slice preparations (Hajos et al., 2009; Ivanov and Zilberter, 2011), prompting calls to study the feedback loop between activity and energy availability (Zilberter et al., 2010). Such approaches may yield new insights into activity that requires high metabolic load, such as the high-frequency gamma activity associated with higher cognitive functions (Kann, 2011). Furthermore, live O_2_ monitoring enables unprecedented insight into metabolic dynamics (Ingram et al., 2014), motivating new models of seizure dynamics (Wei et al., 2014), complementing models of ion concentrations (Cressman et al., 2009). This last application is notable because brain dynamics have been shown to deviate from criticality during seizures (Meisel et al., 2012).

Thus, we argue that since signatures of scale-free dynamics appear to be sensitive to metabolic disturbances, proper understanding of these dynamics should parsimoniously describe the underlying metabolic system to which the dynamics are closely coupled. This allows the metabolic states to tune the neuronal states. More concretely, typical models of the form

(3) $$\dot{x}=f\left(x,\;M,\;t\right)+\xi (t),$$

where *M* are parameters, can be extended to incorporate dynamics for the slow evolution of *M* given by

(4) $$\dot{M}=\epsilon g(x,\;M,\;t),$$

where *ϵ* is a small parameter determining the separation of time scales. This formalism of slow parameter dynamics (not necessarily metabolic) is widely used to model oscillatory systems such as bursting in individual neurons (Izhikevich, 2000), EEG oscillations in anesthesia (Liley and Walsh, 2013; Ching and Brown, 2014), and seizures (Jirsa et al., 2014). The bifurcations involved in such oscillatory transitions are likely different from the critical points responsible for scale-free dynamics, but the core approach is valid for modeling all types of slow parameter changes, and should be applied in studies of neuronal criticality. In our example of post-hypoxic burst suppression, one could envisage three time scales: fast neuronal dynamics on the order of tens of milliseconds, slower dynamics governing activity-dependence within bursts on the order of hundreds of milliseconds to seconds, and very slow dynamics describing the recovery trajectory in and out of burst suppression on the order of tens of minutes. Indeed such a hierarchy of time scales in a phenomenological model successfully explains many features of seizure dynamics (Jirsa et al., 2014). On slower time scales still, we expect that another key target for such modeling will be the sleep-wake cycle, which is itself fundamentally tied to slow homeostatic processes and known to exhibit temporally-varying signatures of criticality (Meisel et al., 2013; Priesemann et al., 2013).

More broadly, all mechanisms for slow parameter modulations are tightly constrained by the need for the brain to optimize the use of its resources. This view has been extraordinarily successful in explaining the structure of brain networks in terms of minimizing wiring costs (Bullmore and Sporns, 2012), yet has been used only sparingly to study large-scale brain dynamics. The brain evolved under the constraint of finite resources, so understanding how this constraint shapes brain dynamics will likely tell us more about the specific resource constraints, the resulting dynamics, and how the brain may be organized to circumvent these restrictions. Most attention thus far has been devoted to overall activity levels (Attwell and Laughlin, 2001), and even then most of the brain's energy expenditure remains unexplained (Raichle, 2006; Buzsáki et al., 2007). We hypothesize that resource constraints not only underpin activity-dependent modulations on micro- and meso-scales, but collectively act to keep the brain near a critical point on the macro-scale. That is, optimizing the balance between the brain's competing needs of being active while not squandering its energy supplies seems consistent with self-organization to a critical point. Failures of this balance lead to neurological disorders (Meisel et al., 2012; Roberts et al., 2014), demonstrating that studying pathological activity—particularly in metabolically-demanding states—enables better understanding of healthy brain states.

In sum, these considerations suggest new unifying principles across the spectrum of criticality in neural systems as well as new means of disambiguating between competing causal mechanisms. Crucially, this approach also suggests a means of integrating data from emerging technologies that combine electrical, hemodynamic, and metabolic imaging—a major upcoming challenge for neuroscience.

### Conflict of interest statement

The authors declare that the research was conducted in the absence of any commercial or financial relationships that could be construed as a potential conflict of interest.
